# Sex differences in cancer mechanisms

**DOI:** 10.1186/s13293-020-00291-x

**Published:** 2020-04-15

**Authors:** Joshua B. Rubin, Joseph S. Lagas, Lauren Broestl, Jasmin Sponagel, Nathan Rockwell, Gina Rhee, Sarah F. Rosen, Si Chen, Robyn S. Klein, Princess Imoukhuede, Jingqin Luo

**Affiliations:** 1 Department of Pediatrics, Washington University School of Medicine, 660 South Euclid Avenue, St Louis, MO 63110 USA; 2 Department of Neuroscience, Washington University School of Medicine, 660 South Euclid Avenue, St Louis, MO 63110 USA; 3 Department of Medicine, Washington University School of Medicine, 660 South Euclid Avenue, St Louis, MO 63110 USA; 4 Department of Biomedical Engineering, Washington University School of Medicine, 660 South Euclid Avenue, St Louis, MO 63110 USA; 5 Department of Surgery, Washington University School of Medicine, 660 South Euclid Avenue, St Louis, MO 63110 USA

**Keywords:** Sex differences, Cancer, Epigenetics, Senescence, Immunity, Metabolism, p53, Tumor Suppressor, Angiogenesis

## Abstract

We now know that cancer is many different diseases, with great variation even within a single histological subtype. With the current emphasis on developing personalized approaches to cancer treatment, it is astonishing that we have not yet systematically incorporated the biology of sex differences into our paradigms for laboratory and clinical cancer research. While some sex differences in cancer arise through the actions of circulating sex hormones, other sex differences are independent of estrogen, testosterone, or progesterone levels. Instead, these differences are the result of sexual differentiation, a process that involves genetic and epigenetic mechanisms, in addition to acute sex hormone actions. Sexual differentiation begins with fertilization and continues beyond menopause. It affects virtually every body system, resulting in marked sex differences in such areas as growth, lifespan, metabolism, and immunity, all of which can impact on cancer progression, treatment response, and survival. These organismal level differences have correlates at the cellular level, and thus, males and females can fundamentally differ in their protections and vulnerabilities to cancer, from cellular transformation through all stages of progression, spread, and response to treatment. Our goal in this review is to cover some of the robust sex differences that exist in core cancer pathways and to make the case for inclusion of sex as a biological variable in all laboratory and clinical cancer research. We finish with a discussion of lab- and clinic-based experimental design that should be used when testing whether sex matters and the appropriate statistical models to apply in data analysis for rigorous evaluations of potential sex effects. It is our goal to facilitate the evaluation of sex differences in cancer in order to improve outcomes for all patients.

## Overview of sex differences in cancer

Sex differences are evident in tumor incidence and mortality throughout the world, across a wide age range, and many different cancer types. Mo,st cancers with a clear sex difference affect males more than females, with incidence rates ranging from 1.26:1 to 4.86:1 (Table 1). These male predominant cancers include hematological malignancies, as well as cancers of the bladder, colon, skin, liver, and brain [1, 2]. Males not only develop cancer more often but are also more likely to die from their disease [3–5]. To date, sex differences have also been demonstrated in rates and patterns of metastasis, expression of prognostic biomarkers, and response to different types of therapies in several different cancer types [6–8]. Despite this overwhelming evidence, sex differences have not been consistently considered when studying cancer, designing therapies, or constructing clinical trials. In part, this is driven by incomplete recognition of the varied mechanisms that contribute to sexual differentiation and an overemphasis on the role that circulating sex hormones plays in mediating sex differences in cancer.

**Table 1:** Cancers with a clear sex disparity in age-adjusted incidence rates per 100,000 according to SEER explorer incidence data

Cancer type	Male average incidence rates	Female average incidence rates	Male:female incidence ratio
Oropharynx and tonsil	4.18	0.86	4.86
Larynx	5.2	1.1	4.73
Esophagus	7.34	1.74	4.22
Urinary Bladder	35.24	8.74	4.03
Mesothelioma	1.58	0.4	3.95
Lip	1.02	0.32	3.19
Liver and intrahepatic bile duct	13.58	4.72	2.88
Tongue	5.22	1.92	2.72
Oral cavity and pharynx	17.02	6.38	2.67
Floor of mouth	0.68	0.3	2.27
Kidney and renal pelvis	22.16	10.92	2.03
Myelodysplastic syndromes	6.2	3.26	1.9
Stomach	10	5.36	1.87
Salivary gland	1.7	1.02	1.67
Leukemia	18.06	10.9	1.66
Melanoma of the skin	28.78	17.46	1.65
Myeloma	8.7	5.58	1.56
Non-Hodgkin lymphoma	23.9	16.22	1.47
Gum and other mouth	1.84	1.28	1.44
Soft tissue including heart	4.14	2.92	1.42
Brain and nervous system	7.52	5.36	1.4
Small intestine	2.74	2.06	1.33
Eye and orbit	1.02	0.78	1.31
Colon and rectum	44.28	33.98	1.3
Lung and bronchus	63.08	48.94	1.29
Pancreas	14.66	11.48	1.28
Hodgkin lymphoma	3	2.38	1.26
Anorectal	1.56	2.22	0.7
Gallbladder	0.9	1.5	0.6
Thyroid	8.04	23.26	0.35
Breast	1.24	127.5	0.01

Incidence rates were calculated by averaging age-adjusted incidence rates per 100,000 from the last 5 years (2012–2016). Male:female ratio was calculated by dividing the male incidence rate by the female incidence rate, both shown in the table. The geometric mean of the male/female incidence ratio is 1.5

Circulating estrogen, progesterone, and testosterone, undoubtedly, contribute to the genesis and progression of some cancers. Breast and prostate cancer, for example, clearly respond strongly to circulating sex hormones [9–11]. Additionally, estrogen has been shown to be anti-tumorigenic for liver and colon cancer (which show a male predominance), and pro-tumorigenic for meningiomas and thyroid cancer (which show a female predominance). However, the molecular basis for the sex disparity in most cancers is still undefined [12–42]. For most cancer types, the magnitude of sex differences in incidence and severity do not parallel the age-dependent changes in circulating sex hormone abundance [36, 43]. Thus, circulating sex hormone actions cannot account for all sex differences in cancer, and acute hormone-independent cancer mechanisms remain to be fully determined.

Sex differences in cancer as well as in normal physiology, arise through sexual differentiation, a process involving genetics and epigenetics, in addition to acute sex hormone actions. Consequently, males and females differ in their rates of growth [44], myelination [45], immunity [46, 47], cardiovascular function [48], systemic metabolism [49], aging, and wound healing [50]. In this light, it should come as no surprise that sexual differentiation affects cancer incidence, response to treatment, and survival.

In this review, we will focus on specific cancer core mechanisms to illustrate how sexual dimorphisms in basic biological functions influence cancer biology, and might impact response to treatment. In some instances, we will focus on brain tumors for the following reasons: The geometric mean value for the sex differences ratio (M/F) in cancer incidence is approximately 1.5:1 (Table 1). The bias in glioblastoma (GBM) incidence is approximately 1.6:1 [51]. We expect that if there are adaptations to make in our science to best incorporate common mechanisms underlying sex differences in cancer, they will be most easily identified in a cancer like GBM, that occurs with a mean sex bias in incidence and for which there are data that span the scales of oncology research from the cellular to the patient level [21, 35, 52–59]. We conclude with a discussion of the rigorous statistical approaches for studying sex effects in the laboratory and the clinic (Table 2).

**Table 2:** Content outline

1. Overview of sex differences in cancer	
Table 1 Cancers with a clear sex disparity in age-adjusted incidence rates per 100,000 according to SEER explorer incidence data	
2. Epigenetics	
2.1: Epigenetics and cancer	
2.2: Sex differences in epigenetics	
2.3: Implications for targeting epigenetics	
Fig. 1 Sex-specific epigenetic programming may contribute to differential barriers to tumorigenesis in males and females	
3. Metabolism	
3.1: Metabolism and cancer	
3.2: Sex differences in metabolism	
3.3: Implications for targeting metabolism	
Fig. 2 Sex differences in metabolic pathways may contribute to sex differences in cancer development	
4. p53	
4.1: p53 and cancer	
4.2 Sex differences in p53	
4.3: Implications for targeting p53	
5. Cellular senescence	
5.1: Senescence and cancer	
5.2: Sex differences in senescence	
5.3: Implications for targeting senescence	
Fig. 3 Sex differences in senescence and SASP may contribute to the increasing sex disparity in cancer incidence with age	
6. Immunity	
6.1: The immune system in cancer	
6.2: Sex differences in the immune system	
6.3: Implications for immunotherapy	
Fig. 4 Sex differences in immune cells affecting cancer development	
7. Angiogenesis	
7.1: Angiogenesis and cancer	
7.2: Sex differences in angiogenesis	
7.3: Implications for targeting tumor angiogenesis	
Fig. 5 Sex differences in endothelial cells, endothelial progenitor cells, circulating angiogenic factors, and sex hormones contribute to sex differences in tumor angiogenesis	
8. Statistical considerations	
Fig. 6 Visualizing statistical interaction	
9. Perspectives and significance	

## Epigenetics

### Epigenetics and cancer

Over the past two decades, epigenetic dysregulation has emerged as a critical mechanism of cancer initiation and adaptation. We now recognize that essentially all cancer hallmarks that can be acquired through genetic mutation can similarly be achieved through epigenetic mechanisms. This can involve aberrant activation or silencing of specific loci, or global remodeling of the epigenetic landscape. Genes encoding epigenetic readers, writers, and erasers, as well as histone proteins themselves, are frequently mutated in human tumors [60–64], drawing a direct link between epigenetic dysregulation and tumorigenesis. Cellular differentiation is a process encoded by progressive layers of epigenetic restriction [65], and disruption of the normal epigenetic landscape can enable cancer cells to reactivate developmental programs and acquire features resembling stem cells [66, 67]. Epigenetic mechanisms also contribute to intra-tumor heterogeneity and therapeutic resistance [68–70]. Thus, targeting tumor epigenetics may be a strategy for improving treatment response [68, 71–73]. In this section, we will review the evidence that the epigenetic landscape of male and female cells differs, that this is a critical mechanism by which sexual dimorphism is established, and that this fundamental divergence in male and female biology, present in every cell of the body, has important implications for cancer risk and treatment.

### Sex differences in epigenetics

Numerous studies have identified consistent sex differences in the epigenetic landscape in multiple tissues, spanning all ages, and across species. The most extensively profiled sexually dimorphic epigenetic mark is DNA methylation. Sex-specific methylation patterns have been observed in blood [74–77], placenta [78], liver [79–82], pancreas [83], muscle [84], heart [81], and brain [81, 85–89]. Sex differences in histone modifications have also been described, although thus far only in mouse brain [90, 91]. Since the majority of mechanistic studies on epigenetics and sexual differentiation have been performed in the brain, this will be our focus here; however, since sex differences in epigenetics are found throughout the body, it is likely that many of these mechanisms will apply to tissues more broadly.

Sexual dimorphism in the brain has been recognized since the 1950s and is understood to be largely determined by exposure to gonadal hormones during a critical window of in utero development. Early studies in rodents found that an injection of testosterone administered in utero, or during the perinatal period, results in permanent masculinization of adult behavior in females [92, 93], and evidence that this model also applies to humans is supported by case studies of disorders of sex development [94, 95]. The finding that testosterone exposure resulted in a long-lasting and stable patterning of sexual differentiation led to the hypothesis that epigenetic programming may underlie sex differences, and a number of studies in the past decade have provided mechanistic evidence for this theory.

Neonatal female rats have higher levels of the DNA methyltransferase Dnmt3a in the amygdala compared to males, and treating with testosterone significantly decreased this expression [96], indicating it is an important regulator of DNA methylation. In agreement with this, neonatal female rats also had higher methylation levels in the promoter of the estrogen receptor (ER)-α gene, compared to both males and females masculinized by gonadal hormone exposure [97]. Genome wide methylation surveys of the striatum and bed nucleus of the stria terminalis/preoptic area (BNST/POA), a known sexually dimorphic brain region, showed that female mice treated with testosterone on postnatal day 0 had an altered methylation pattern that resembled that of males, when they were profiled at postnatal day 60 [85]. In 2015, Nugent et al. demonstrated that gonadal steroid exposure during development decreased the activity of DNA methyltransferases (DNMTs) in the POA, lowering DNA methylation levels in females to levels equivalent to males. Knocking out *Dnmt3A* or pharmacologically inhibiting Dnmts masculinized sexual behavior in females, even when treatment was given outside the critical window [98]. Additionally, treatment with DNMT inhibitors reverses some anatomical and functional sex differences in the POA [99]. Together, these studies suggest that DNA methylation actively suppresses masculinizing genes in order to maintain brain feminization, and that this depends on levels of gonadal hormones during development. Intriguingly, when embryonic neural stem cells (eNSCs) were treated with testosterone in vitro, it resulted in a global decrease in DNA methylation in both XX and XY cells [100]. A similar result was reported for DNA methylation in liver, in which males were hypomethylated compared to females, and this was dependent on testosterone exposure [82]. These studies indicate that sexual differentiation involves sex-specific regulation of DNA methylation.

Differences in male and female methylation patterns may have important implications for cancer development. One epigenetic change recognized in many cancers, though with some exceptions (notably isocitrate dehydrogenase (IDH)-mutant gliomas [101]), is a propensity for global hypomethylation [102, 103]. DNA hypomethylation is associated with increased cancer malignancy, and mutations in *Dnmts* are cancer promoting in multiple mouse models. Broad regions of hypomethylation (both DNA and histone) are believed to contribute to dedifferentiation and the cancer stemcell-like state, and to increase epigenetic plasticity [62]. Another scenario in which cells reacquire a stem cell phenotype is through reprogramming to induced pluripotent stem cells (iPSCs), a process that has some parallels to cancer evolution [67]. During reprogramming, DNA methylation marks associated with cell type-specific differentiation are erased, and reprogramming efficiency can be enhanced by the inhibition of DNMTs [104]. Thus, male- and female-specific methylation patterns could influence the ability of cancer cells to adopt a stem cell-like phenotype.

Sex differences in histone modifications also underlie sexual differentiation of the brain. Matsuda et al. found that there were sex differences in histone acetylation levels of the ERα and aromatase promoters, two genes essential for masculinization, during the critical period. Inhibiting histone deacetylases (HDACs) at postnatal day 0/1 resulted in decreased male sexual behavior, suggesting that histone deacetylation is required for proper sexual differentiation [105]. HDAC inhibitors also eliminated anatomical sex differences in the BNST [106]. Treating eNSCs with testosterone in vitro led to a global increase in histone H3 acetylation in daughter lineages, supporting the hypothesis that gonadal hormones can exert stable effects on the genome via histone modifications [100]. Of note, upregulated genes in both XX and XY eNSCs treated with testosterone were highly enriched for pathways involved in nucleosome organization, nucleosome assembly, and chromatin assembly, suggesting that testosterone-mediated transcriptional changes could drive downstream epigenetic reorganization [100]. Together, these studies provide strong evidence that gonadal steroid exposure during the critical period mediates sexual differentiation of the brain via epigenetic mechanisms.

Gonadal hormone exposure is not the only mechanism by which epigenetics can diverge in males and females. In preimplantation embryos, hundreds to thousands of genes differ in expression between the sexes [107–110], despite the fact that gonadal differentiation has yet to occur. The basis of sexual dimorphism in these early embryos is the unique complement of sex chromosomes in male (XY) and female (XX) cells. One of the most striking differences in male and female epigenetics is the inactivation of the additional X-chromosome in female cells. This inactivation is orchestrated by the long non-coding RNA (lncRNA) *XIST*, which mediates chromosome-wide silencing through histone deacetylation and subsequent enrichment of repressive chromatin marks [111]. This results in a dense, highly stable, heterochromatic region unique to female cells. Removal of these heterochromatic marks, and reactivation of the X-chromosome, is one of the steps that takes place during reprogramming to iPSCs in mouse cells [112], though the status of the inactive X is more complicated in human iPSCs [113, 114]. As mentioned above, reprogramming to iPSCs has some parallels to cancer dedifferentiation. Intriguingly, generation of iPSCs from mouse embryonic fibroblasts is more efficient when using male cells than when using female cells, and it is speculated that this may be due to an X-chromosome reactivation barrier [115]. Furthermore, there is evidence that X inactivation is lost in some female cancers, through either mitotic errors or epigenetic dysregulation and reactivation [116, 117]. Whether X-chromosome reactivation dynamics contribute to female protection in cancer has not been investigated.

*XIST* and other lncRNAs of the X inactivation center (XIC) may have more direct roles in tumor risk as well. *XIST* appears to be both tumor-promoting and tumor-suppressive, depending on cancer type and context [118]. Surprisingly, two meta-analyses of *XIST* in cancer identified no association between *XIST* and sex/gender, but did find that high levels were associated with poor overall survival [119, 120]. Although *XIST* is not normally expressed in XY cells, these studies suggest that aberrant regulation of this lncRNA can occur in both male and female tumors. *FTX*, another lncRNA involved in X inactivation, has been identified as a putative tumor suppressor in hepatocellular carcinoma (HCC). It is expressed at higher levels in tumors from female patients, positively correlates with survival, and inhibits HCC cell proliferation and invasion [121]. Additional XIC lncRNAs, such as *JPX* [122] and *TSIX* [123] may also have roles in cancer initiation and progression. Because expression of lncRNAs involved in X inactivation differs in normal XX and XY cells, this could contribute to sex differences in cancer risk. In addition to the lncRNAs of the XIC, multiple other lncRNAs, located throughout the genome, have been identified as sex-biased in expression [110, 124, 125]. Functions of lncRNAs include modifying chromatin state and regulating gene expression [126]; thus, differences in even a small number of lncRNAs could have wide ranging effects. As with protein-coding genes, lncRNAs can act as either tumor suppressors or tumor promoters, and are now recognized to be frequently dysregulated in cancer [127–131].

LncRNAs also have important roles in regulating imprinting. In genomic imprinting, a gene is preferentially expressed from either the maternal or paternal allele [132]. Since both male and female embryos inherit a full set of autosomes from their mother and father, the majority of imprinted regions do not differ between the sexes. However, there is evidence that, at least in brain, the sex of the offspring can affect imprinting, with some autosomal loci imprinted in one sex but not the other [110, 133]. The greatest differences in imprinting emerge from the X-chromosome. Male embryos inherit only the maternal X, while female embryos inherit both a maternal and paternal X [132]. Differences in the phenotype of XO girls who inherit a paternal (X_p_) vs. a maternal (X_m_) X-chromosome, suggest that X-chromosome imprinting does impact development, particularly in the brain [132, 134]. Intriguingly, one study found a trend towards increased brain volume in X_m_O girls, compared to X_p_O girls [134], suggesting potential roles of X imprinted genes on regulating growth. However, no studies have examined if cancer rates differ in X_m_O or X_p_O women, or if imprinted X genes contribute to sex differences in cancer risk.

Prior to X-chromosome inactivation (XCI), female embryos are exposed to a double dose of X-chromosome genes, while male embryos get a single X dose plus a much smaller number of Y-chromosome genes. However, even after X-chromosome inactivation, a percentage of genes on the X (15% in humans [135] and 3% in mouse [136]) escape inactivation, and are expressed at higher levels in females. Differences in X and Y gene expression appear to exert broad downstream effects, resulting in genome-wide transcriptional differences [100, 110, 137]. One mechanism by which sex chromosomes may exert broad regulatory effects is through epigenetic regulation. A number of important epigenetic modifiers are located on the X-chromosome [138], including lysine demethylases *KDM5C* and *KDM6A* (*UTX*). Both *KDM5C* and *KDM6A* are known to escape X-chromosome inactivation, are expressed at higher levels in females, and do not appear to be fully compensated by their Y-chromosome paralogues *KDM5D* and *UTY* [139–141]. *KDM6A* and *KDM5C* are two of several putative tumor suppressors on the X-chromosome proposed to contribute to decreased cancer risk in females. These genes, along with *ATRX, DDX3X*, *CNKSR2*, and *MAGEC3* are more frequently mutated in male tumors [142], presumably because females have a second copy to compensate for any loss of function mutations via XCI escape*.* KDM6A in particular has been found to contribute to sex differences in bladder cancer, where it acts as a tumor suppressor in XX individuals [143]. It has also been identified as a tumor suppressor in B cell lymphoma [144], T cell acute lymphoblastic leukemia [145], and pancreatic cancer [146]. In addition to differences in the expression levels of protein-coding genes located on the sex chromosomes, lncRNAs [147] and microRNAs (miRNAs) can also escape X inactivation and differ in expression between the sexes. The X-chromosome contains an unusually high number of miRNAs, 118 compared to an average of 40–50 on the autosomes [148]. These miRNAs are regulators of a diverse array of processes, many of which are relevant to cancer [131, 149, 150].

Finally, in addition to both gonadal hormone and sex chromosome effects, other complex mechanisms may contribute to sex differences in epigenetics. For example, the enzymes that catalyze chromatin modifications require metabolites as both cofactors and substrates [151, 152], and to add further complexity, some metabolites can act as inhibitors of epigenetic enzymes; the ketone body d-β-hydroxybutyrate is an HDAC inhibitor [153], and the oncometabolite 2-hydroxyglutarate (2-HG) acts as a competitive antagonist to α-KG-dependent demethylases [154]. As detailed below, metabolism differs fundamentally in males and females, providing another potential source for epigenetic sex differences.

**Fig. 1:**
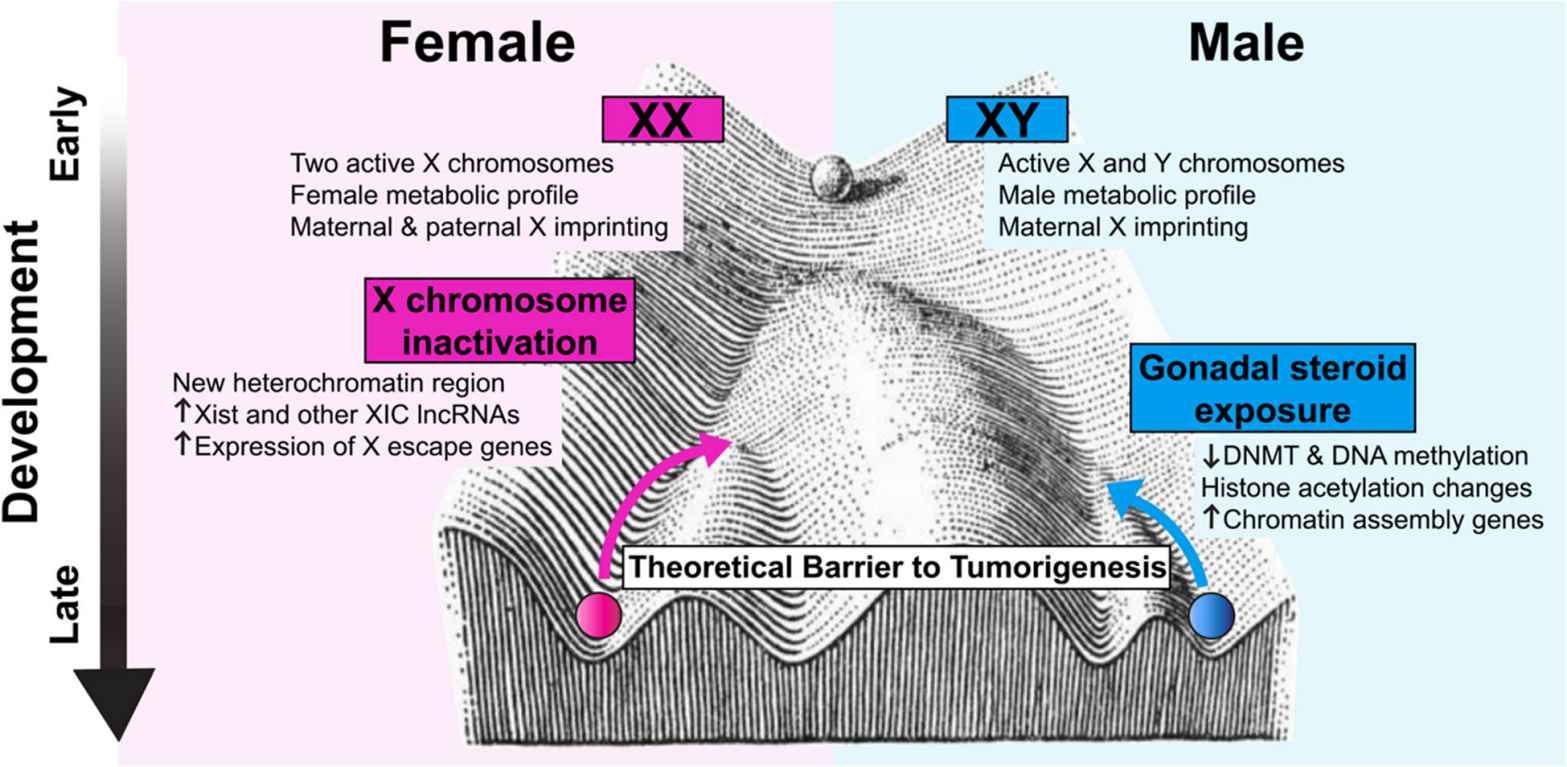
Sex-specific epigenetic programming may contribute to differential barriers to tumorigenesis in males and females. Divergence in male and female epigenetics begins from the moment of fertilization, driven by differences in sex chromosome gene expression and metabolic profiles between XX and XY embryos. Throughout development, additional processes, such as X-chromosome inactivation and gonadal steroid exposure, further differentiate the sexes. The unique epigenetic landscapes of male and female cells may contribute to sex differences in the barrier to tumorigenesis, as well as to sex differences in tumor heterogeneity and response to treatments—both conventional and epigenetic targeted

Together, these varied mechanisms lead to fundamental differences in the epigenomes of male and female cells that likely contribute to sex differences in the cell intrinsic barrier to malignant transformation (Fig. 1).

### Implications for targeting epigenetics

Given the central role of epigenetic dysregulation in cancer initiation, progression, and therapeutic resistance, it is no surprise that drugs targeting epigenetic regulators are emerging as promising cancer therapeutics [73]. There is already evidence that treating with DNMT inhibitors can affect sexually dimorphic epigenetic marks, even when given after sexual differentiation is complete [98], and a recent study showed that combination treatment with an EZH2 inhibitor and HDAC inhibitor at sub-therapeutic levels disrupted X-chromosome inactivation in normal human female fibroblasts [155]. Furthermore, how sex differences in epigenetics interact with epigenetic dysregulation in cancer is currently not well understood. Thus, it will be critical to evaluate clinical efficacy and side effects of epigenetic therapeutics in both sexes separately.

## Metabolism

### Metabolism and cancer

**Fig. 2:**
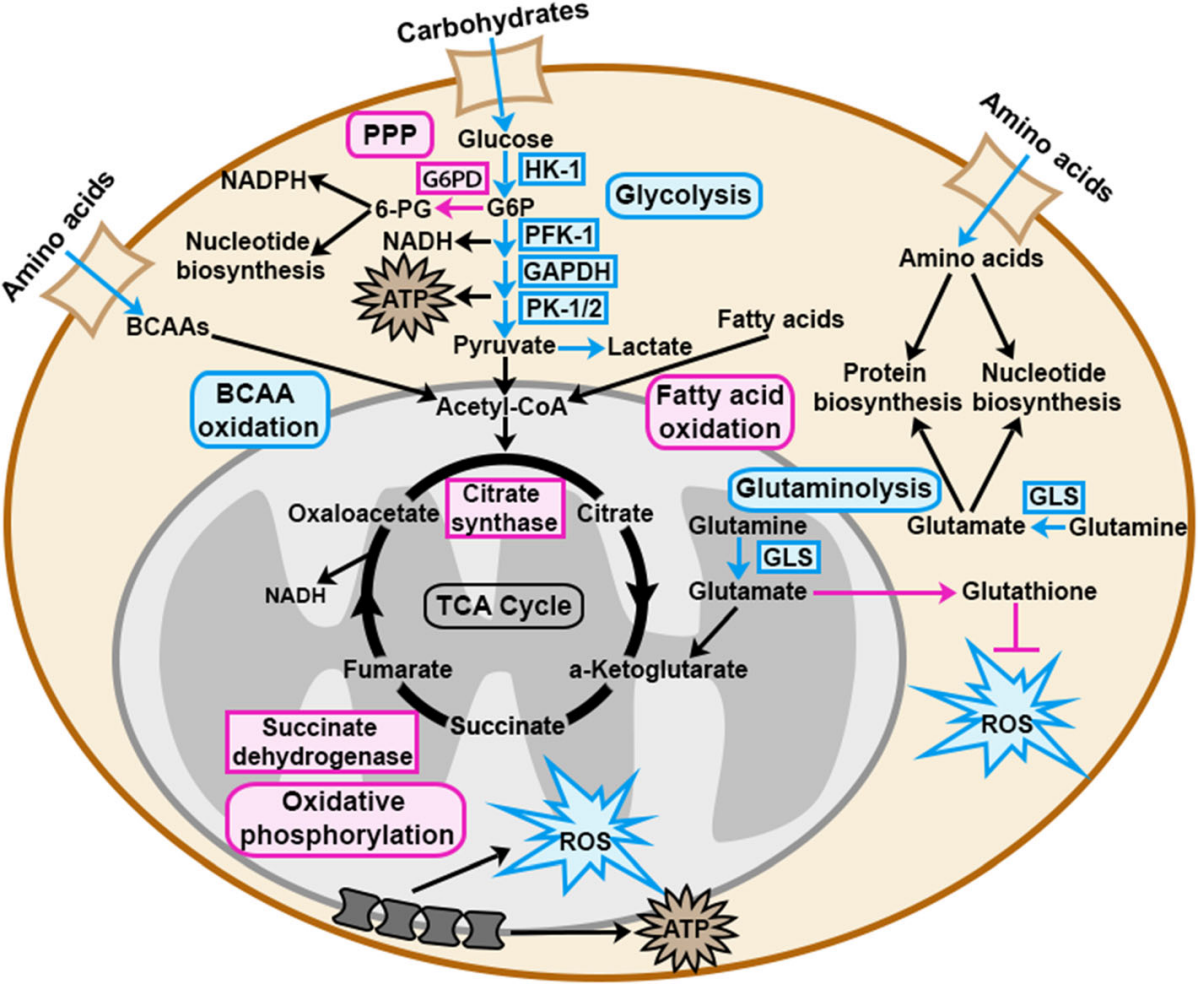
Sex differences in metabolic pathways may contribute to sex differences in cancer development, progression, and treatment response. During development and throughout adulthood, males and females utilize nutrients differently. Males favor carbohydrate and amino acid metabolism, while females favor fatty acid metabolism. Furthermore, female mitochondria produce less ROS despite their higher mitochondrial activity. These fundamental sex differences in nutrient utilization and mitochondrial activity may contribute to sex differences in metabolic reprogramming in cancer cells, which is important during cancer development, cancer progression, and response to anti-cancer treatment. This schematic focuses on metabolic pathways that are known to be sexually dimorphic and important in cancer. Metabolic pathways, metabolites, and metabolic enzymes shown in blue or pink are known to be higher in males or females respectively

Sex differences in metabolism have been extensively reported during development, adulthood, and in certain diseases, such as obesity and diabetes. Even though metabolic reprogramming is an essential process in cancer, sex differences in cancer metabolism have not yet been considered. Cancer cells undergo metabolic reprogramming to optimize their biomass and energy production, which allows them to proliferate rapidly. Metabolic reprogramming involves increased uptake of nutrients, increased catabolic metabolism to produce ATP, and increased anabolic metabolism to produce biomass. The latter also requires cancer cells to reprogram mitochondrial metabolism, as many anabolic processes take place in the mitochondria. Furthermore, cancer cells must be able to adapt to the unique metabolic stressors that accompany cancerous growth, including shortage of nutrients, insufficient oxygen supply, and an increase in oxidative stress. Metabolic reprogramming in cancer cells is heavily reviewed elsewhere [156, 157]. In this section, we will focus on three key components of metabolic reprogramming in cancer: (i) nutrient utilization, (ii) mitochondrial activity, and (iii) reactive oxygen species (ROS) regulation. We will review the literature describing sex differences in these metabolic processes (summarized in Fig. 2) and discuss how these sex differences might contribute to sex disparities in cancer.

### Sex differences in metabolism

Sex differences in metabolism are evident at every stage of life and at the organismal, tissue, and cellular levels. Across multiple species, including mice, bovine, birds, and humans, male embryos grow faster than female embryos and exhibit concordant differences in nutrient utilization and energy consumption [158–163]. In humans, pyruvate and glucose uptake, as well as lactate production, are significantly higher in male embryos [158], and the presence of male fetuses has been associated with elevated maternal fasting plasma glucose [164, 165]. In cows, a high concentration of glucose selectively blocks development of female embryos during the morula to blastocyst transition [166–168]. Furthermore, glucose metabolism is twice as high in male compared with female bovine embryos [169], and glycolytic genes such as Hexokinase-1, Phosphofructokinase-1, Pyruvate kinase-1/2, GAPDH, and Glucose transporter-1 are all more highly expressed in male bovine embryos [170]. In contrast, female bovine embryos exhibit higher pentose phosphate pathway activity [169]. This sex bias is at least partially due to sex chromosome complement. The pentose phosphate pathway genes glucose-6-phosphate dehydrogenase (G6PD) and O-linked N-acetylglucosamine (GlcNac) transferase (OGT) are located on the X-chromosome and were found to be more highly expressed in female mouse and bovine embryos respectively [171, 172]. In human adults, metabolites of carbohydrate pathways such as glycolysis, gluconeogenesis, and pyruvate, and fructose, mannose, and sucrose metabolism are enriched in male serum compared to female serum [173]. In summary, male embryos exhibit higher glucose uptake and glycolytic activity than female embryos, while female embryos favor the pentose phosphate pathway. In adults, carbohydrate metabolites are enriched in serum of males as compared to females.

Sex differences in amino acid utilization have also been reported. Increased amino acid intake during the first week of life was associated with a significant short-term improvement in weight gain in male, but not female, low birth weight infants [174, 175]. In human adults, male serum is enriched for amino acid metabolites, including branched chain amino acid metabolites, glutamate metabolites, lysine metabolites, phenylalanine and tyrosine metabolites, cysteine and methionine metabolites, and tryptophan metabolites [173, 176]. Furthermore, exercising adult men oxidize significantly higher levels of the branched-chain amino acid leucine than exercising women [177]. Additionally, the male rat brain contains higher levels of glutamate and a higher ratio of glutaminase/glutamine synthetase, suggesting that brains of male rats utilize more glutamate from glutamine [178]. Even in moths, adult males oxidize certain amino acids (leucine, phenylalanine, and glycine) at a higher rate than females [179]. Together, these data show that men exhibit higher levels of most amino acid metabolites in their serum, and for those amino acids tested, males exhibit higher rates of metabolism than females, across different ages and species. Whether this is true for all amino acids has yet to be determined.

While the above studies indicate that males exhibit higher rates of glucose and amino acid utilization, females appear to favor lipid substrates for energy metabolism. Upon exercise and during fasting, women utilize more fatty acids while men favor carbohydrate utilization [180]. Numerous publications have shown that lipid metabolism is sexually dimorphic in humans and rodents [181]. Briefly, females exhibit higher rates of lipid biosynthesis than males [182], and enhanced fatty acid clearance in muscle tissue compared to males [183, 184], female rat livers incorporate more fatty acids into glycerolipids and fatty acid oxidation products [185], female rodents and humans are more resistant to free fatty acid-induced insulin resistance [183, 186], and sex differences in expression and activity of lipid metabolism enzymes, such as lipoprotein lipase, are well established [187].

As males and females differ in their fundamental nutrient utilization for metabolism, it is likely that mechanisms of metabolic reprogramming in cancer differ between men and women. For example, reprogramming of amino acid and carbohydrate metabolism may be required for transformation of male cells, while reprogramming of fatty acid metabolism may be required for transformation of female cells. Support for important sex differences in cancer metabolism comes from two publications. In the first, glycolytic gene overexpression in low-grade glioma was shown to correlate with decreased survival in men but not women [188]. In the second, high visceral fat quantity correlated with decreased survival in women with renal cell carcinoma but not men [189]. Thus, sex differences in metabolic reprogramming might contribute to different thresholds for cellular transformation, cancer progression, and outcome, and might therefore contribute to the sex disparity in cancer susceptibility and outcome.

For decades, the primary focus in cancer metabolism research has been on increased nutrient uptake and utilization through aerobic glycolysis (Warburg effect). Today, numerous studies have shown that cancer cells also exploit the mitochondrial tricarboxylic acid cycle (TCA cycle) and oxidative phosphorylation [190, 191]. Mitochondria are a primary source of reactive oxygen species (ROS), and ROS production and consumption through mitochondrial antioxidant pathways, such as glutathione oxidation, are required for well-balanced regulation of ROS levels [192]. Mitochondria exhibit a strong sex-specific behavior as they are exclusively maternally inherited. A vast body of literature describes tissue and cell-specific sex differences in mitochondria morphology, function, and oxidative stress regulation [193]. Here, we will focus specifically on sex differences in the brain; however, since sex differences in mitochondria have been reported in tissues throughout the body, including liver, cerebral arteries, white and brown adipose tissue, pancreas, muscle, and heart, our discussion likely applies to multiple cancer types.

In rat brain, females exhibit higher mitochondrial protein content and higher mitochondrial activity (higher electron transport chain enzyme activity and respiration rate) [194]. These sex differences gradually increase during aging [195]. In concordance with these findings, female mice exhibit an enhanced respiration rate in brain tissue compared to age-matched male mice [196, 197]. Additionally, mitochondria isolated from female mouse brains show higher electron transport chain activity and ATP production than male mitochondria [198]. In contrast, isolated male mitochondria exhibit a higher calcium retention capacity, which can augment ATP production by altering the activity of calcium-sensitive mitochondrial matrix enzymes [199]. Furthermore, in vitro cultures of rat cortical astrocytes under low oxygen conditions showed greater respiratory capacity in male, compared to female astrocytes [200]. In human brains, activity of the mitochondrial enzymes citrate synthase, succinate dehydrogenase, and mitochondrial reductase were significantly higher in females, suggesting a higher mitochondrial activity in women compared to men [201]. Sex differences in mitochondrial biogenesis have also been reported but are inconsistent. In the mouse brain, mitochondrial biogenesis is greater in females [197], despite evidence for increased expression of key regulators of mitochondrial biogenesis in males [202]. In contrast, female rat brains have lower mitochondria content than male brains [194], and this sex difference increases with age [195].

Sex differences in mitochondrial activity suggest that ROS accumulation, ROS regulation, and sensitivity to ROS are also sexually dimorphic, as ROS is generated in the mitochondria during oxidative phosphorylation. Indeed, despite their higher respiratory activity, female mouse brains accumulate significantly lower levels of ROS compared to their age-matched male counterparts [196, 197]. This is also true in rats. A study by Borras et al. showed that female rats exhibit significantly higher expression of antioxidant enzymes, such as glutathione peroxidase and superoxide dismutase. Consequently, female rats exhibit lower levels of ROS, despite their increased respiratory activity, resulting in a better oxidative balance [203]. These findings were complemented by a study from Guevara et al. who showed that antioxidant enzyme levels were higher in female mitochondria [194]. Oxidative damage accumulates in the brain throughout aging. However, in rat brains, the aging effect was less marked in females, which accumulated less oxidative damage as they aged [195]. In humans, biomarkers of oxidative stress are lower in healthy young women than in age-matched men [204]. Together, this data suggests that female mitochondria exhibit a better oxidative balance, accumulating less ROS despite their higher respiratory rate. Thus, female mitochondria appear to be better optimized than male mitochondria.

ROS can be both pro-tumorigenic and anti-tumorigenic. Certain oncogenic mutations, such as mutations in the Ras pathway, lead to increased ROS levels. In turn, ROS levels can stimulate growth factor signaling pathways, driving tumor development through promotion of proliferation, angiogenesis, invasion, and metastasis [205]. Excessive ROS levels, however, can be cytotoxic, as they can induce apoptosis, autophagy, and necroptosis [206]. Since male mitochondria accumulate more ROS than female mitochondria, male cells may have a higher risk of carcinogenesis than female cells. Furthermore, it has been shown that, with age, ROS levels accumulate more in males than in females [195, 204]. A similar correlation can be found with cancer incidence. With age, cancer incidence rate increases with a steeper slope in men than in women (see “Senescence” section). Therefore, sex differences in mitochondrial activity and ROS regulation may underlie different thresholds for malignant transformation in men and women and might therefore contribute to sex differences in cancer susceptibility.

### Implications for targeting metabolism

In the last decade, metabolic targeting in cancer has made many advances. However, sex has not been adequately considered as a biological variable in cancer metabolism. Here, we will discuss metabolic treatment approaches that are currently in clinical trials for cancer and why they might have sex-specific effects.

Inhibiting the PI3K/mTOR pathway has been shown to be effective in many cancers, and multiple PI3K/mTOR inhibitors are currently in clinical trials [207, 208]. The PI3K/mTOR pathway is a major regulator of metabolism [209]. Sex differences in metabolic pathways such as glycolysis raise the question of whether the PI3K/mTOR pathway might be sexually dimorphic, and therefore, underlie sex differences in metabolic pathways. In fact, research done in drosophila and murine heart tissue showed that the PI3K/mTOR pathway activity is different in males and females [210, 211], which suggests that targeting this pathway might result in sex-specific responses.

Amino acid metabolism and carbohydrate metabolism have also been targeted in cancer. The effect of the glutaminase inhibitor CB-839 and the effect of a ketogenic diet (low-carbohydrate diet) are both currently being evaluated in clinical trials [212, 213]. Most published data indicate that males utilize more carbohydrate and amino acid substrates for metabolism, while females utilize more fatty acids. Therefore, targeting amino acid and carbohydrate metabolism might have sex-specific effects. Targeting mitochondria to induce apoptosis through increased ROS levels has also been shown to be effective in treating brain cancer in mice, and the mitochondrial-targeting drug dichloroacetate (DCA) has been tested in patients with glioblastoma [214, 215]. Given the large body of literature demonstrating sex differences in mitochondrial activity and ROS accumulation, it is likely that targeting ROS might have different effects in men and women with cancer. Since male mitochondria accumulate more ROS than female mitochondria, male cancers may more readily exhaust their capacity to regulate ROS, and consequently, may respond to ROS elevating treatments more robustly than female cancers.

Among the notable recent discoveries in cancer genome-wide sequencing was the discovery of the isocitrate dehydrogenase 1 and 2 (IDH1/2) R132H mutation. IDH1/2 is an enzyme in the TCA cycle that catalyzes the conversion of isocitrate to citrate, and its reverse reaction. Mutant IDH1/2 exhibits loss of normal function and gain of aberrant function, in which isocitrate is converted into D-2-hydroxyglutarate (2-HG), an onco-metabolite that has far-reaching pro-tumorigenic effects on epigenetic regulation, DNA repair, and redox state [216, 217]. Due to its importance for mitochondrial biomass production, oxidative phosphorylation, and fatty acid synthesis, disruption of the TCA cycle through mutant IDH1/2 will most likely result in sex-specific effects during tumor development, progression, and treatment response. In concordance, mutant IDH1/2 inhibitors, which are currently in clinical trials for multiple cancers [218] might exhibit sex-specific effects. In glioblastoma, a sex-specific effect of IDH mutations on overall survival has been evaluated in multiple publications [56, 59, 219]; however, the findings are inconsistent and further study will be required to define how interaction between IDH1 mutation and patient sex impacts on survival.

## p53

### p53 in cancer

The tumor suppressor *TP53* (p53) is the most frequently mutated gene in cancer, with mutations occurring across almost all cancer types and in approximately half of all tumors [220, 221]. p53 is best known as a transcription factor that regulates target genes in response to DNA damage or oncogenic stress to induce cell cycle arrest or apoptosis. In this simplified model, p53 inhibits tumorigenesis by arresting or eliminating preneoplastic cells [222]. Loss of p53 function through mutation or alterations in upstream regulators eliminates this barrier, leading to increased proliferation, genomic instability, and the accumulation of new mutations that drive tumorigenesis. However, p53 actually functions at the center of a complex network of pathways, and can produce many phenotypic outcomes depending on the specific stimulus, tissue or cell type, age, and as described below, sex. In addition to proliferation and DNA damage response, p53 plays important roles in metabolism, pluripotency, epigenetics, ROS regulation, chromatin stability, epithelial to mesenchymal transition (EMT) and invasion, inflammation, and the tumor microenvironment [223]. Currently, tumors are most typically bifurcated into p53-WT and p53-mutant groups, but this is an oversimplification. Mutations in p53 can drive many distinct phenotypes depending on the type of mutation (e.g., missense, truncating, frameshift), co-mutations in other genes, and tissue type [223]. Notably, the most common mutations are missense mutations in the DNA binding domain, many of which have been ascribed with distinct gain-of-function abilities that are more oncogenic than complete p53 deletion [221, 224]. Together, this supports that p53 function is context dependent and must be evaluated within each distinct setting to understand its role in cancer. Here, we will review current evidence on sex differences in p53 function and discuss how these sex differences might contribute to the sex disparity in cancer.

### Sex differences in p53

While p53 is best known for its role as a tumor suppressor, it exhibits sex differences in function across many tissues, throughout normal development and aging. During meiosis, p53 monitors germ cells for DNA damage in males and induces necrosis to eliminate mutant spermatocytes. In female germ cells however, this role is carried out by the p53 family members p63 and p73 [225, 226]. p53 also has differential effects on in utero development. p53 null mice appear developmentally normal but exhibit a sex bias in the ratio of male to female progeny. In the absence of p53, female mice develop neural tube defects that are embryonic lethal. This was recently shown to be caused by incomplete X-inactivation and disruptions in X-gene dosage [227]. p53 directly regulates the long non-coding RNA X-inactivation specific transcript (*Xist*), which is a primary effector of X-inactivation, and the loss of p53 decreases *Xist* expression [228].

Sex differences in birth defects in p53 mutant mice may also be related to a sexually dimorphic role for p53 in epigenetic imprinting. Methylation sequencing revealed that female p53 null offspring with birth defects displayed a hypermethylation phenotype for insulin-like growth-factor 2, compared to mice born without birth defects [229], suggesting a female biased role for p53 in monitoring epigenetic marks during development. Work in drosophila also suggests that p53 may be sexually dimorphic in aging. Overexpression of wild type p53 in flies increased the lifespan of males, while decreasing the lifespan of females [230].

An essential function of p53 is the monitoring of DNA damage and the clearance of pre-neoplastic cells by senescence or apoptosis. A growing body of evidence suggests that the cells-of-origin for GBM are oligodendrocyte precursor cells (OPC) that arise in the subventricular zone (SVZ) before migrating to the cortices [231, 232]. Kim and Casaccia-Bonnefil found that as mice mature, the number of neural progenitor cells in the SVZ decreases faster in males than in females; deletion of p53 eliminated this sex difference. Co-treatment of SVZ cells in vitro with radiation and sex hormones revealed that estrogen, but not testosterone, reduced p53 expression and apoptosis in response to DNA damage [233]. This suggests that p53 function in the SVZ is sexually dimorphic during normal development and in response to exogenously induced DNA damage and may contribute to the sex differences in incidence and survival observed in GBM.

Sex differences in p53 function are readily observed in patients with Li-Fraumeni syndrome (LFS). LFS is a familial cancer predisposition syndrome associated with germline mutations in p53 [234, 235]. LFS patients have a 50% risk of cancer before the age of 30, and an over 90% lifetime risk [236, 237]. The most frequent cancer types include breast cancer, soft tissue sarcoma, brain/CNS tumors, bone sarcoma, and adrenocortical carcinoma. Even when controlling for the high rate of female breast cancer, multiple LFS family cohort studies have concluded that female mutant p53 carriers have an increased risk of developing cancer compared to male carriers [236–239]. Importantly, the numbers of mutant p53 carriers were equivalent between males and females, suggesting that males and females have differential responses to the same mutations in p53, leading to greater risk of cancer development in females. However, Oliver et al. contradict these findings [240]. They observed that mutant p53 carriers exhibited a male bias in cancer incidence that matched the male bias in incidence observed in individuals with sporadic p53 mutations. All five studies, however, reported a female bias in adrenocortical carcinoma (ACC) incidence, which is corroborated by data reported by the International Pediatric Adrenocortical Tumor Registry [241]. Pediatric ACC is a rare cancer (0.72/million/year in the USA) and is strongly associated with mutations in p53 [242]. Because many of these patients are prepubescent, these data provide further support that males and females respond differently to p53 mutations in a tissue-specific manner, and that these differential responses cannot be explained by the effects of circulating sex hormones.

The p53 pathway, encompassing p53 and its upstream regulators, is mutated in approximately 84% of primary GBM tumors [243]. In primary astrocytes lacking the tumor suppressor neurofibromin (NF1), p53 loss is sufficient to induce sex differences in transformation, leading to greater proliferation and clonogenic frequency in male cells compared to female cells. Furthermore, this p53 loss contributed to sex differences in in vivo tumorigenesis. When otherwise isogenic male and female astrocytes lacking functional NF1 and p53 were injected into the brains of both male and female mice, the mice injected with male cells were more likely to form tumors, regardless of the sex of the host mouse [35]. This finding was replicated in a second model of GBM, in which NF1 and p53 were knocked-out in utero by injecting clustered regularly interspaced short palindromic repeats (CRISPR)/CRISPR-associated protein 9 (Cas9) constructs into the lateral ventricles of E14 mouse embryos, and electroporating to target progenitor cells in the SVZ. While all mice developed GBM, males developed tumors and succumbed to their disease faster than females [21]. Depletion of the tumor suppressors *RB1*, *CDKN2A* (p16), or *CDKN1A* (p21) in females eliminated this difference, suggesting female astrocytes possess at least partial compensatory mechanisms for cell cycle regulation in the absence of p53 [21, 35], which contribute to the greater barrier to tumorigenesis in these cells.

In addition to the direct effects of p53, some p53 regulators may also contribute to sex differences in cancer. The E3 ubiquitin-protein mouse double minute 2 homolog (Mdm2) is a direct regulator of p53 protein stability [244]. Under normal conditions, Mdm2 binds and ubiquitinates p53 leading to proteasomal degradation [245]. In response to cell stress, p53 is phosphorylated, preventing Mdm2 interaction and subsequent degradation [246]. In many tumors with WTp53, the p53 pathway is suppressed through amplification and overexpression of Mdm2 [247]. *MDM2* SNP 309 is a T/G single nucleotide polymorphism in the promoter of *MDM2* that increases affinity for the transcription factor Sp1, driving increased expression of Mdm2 mRNA and protein [248]. The T/G allele drives an estrogen-dependent increase in cancer risk in females [249]. A recent study by Haupt et al. identified a network of X-linked genes associated with p53 function [250]. They found that mutations in p53 regulatory genes on the X-chromosome were expressed at the mRNA level less frequently in females than in males with the same mutation. This increased rate of non-expressed mutations occurred more frequently in p53 pathway linked-genes than unaffiliated genes, suggesting that females may be able to protect the p53 pathway through selective inactivation of mutant genes on the X-chromosome.

### Implications for targeting p53

p53 has garnered considerable support over the last two decades as a candidate target for cancer treatment. Primarily, attempts at targeting p53 have relied on one of three methods: (1) introduction of exogenous WTp53, (2) inhibition of negative regulators of p53 such as Mdm2, or (3) small molecules that can force mutant p53 into a wild type conformation with normalized function [251, 252]. Each of these methods is based on the observation that WTp53 is induced in response to oncogenic stress, so that reactivation of the p53 pathway may slow or eliminate cancer. Given the growing body of evidence for sex differences in p53 function, p53 regulators, and tumor initiation and progression, preclinical and clinical studies focused on targeting p53 should be powered to identify sex differences in treatment response.

## Cellular senescence

### Senescence and cancer

Cellular senescence is the process of permanent cell cycle arrest that occurs in response to cellular aging or DNA instability. Senescence primarily acts as a tumor suppressor mechanism, by preventing continued proliferation of damaged, potentially tumorigenic cells [253, 254]. Despite cell cycle exit, senescent cells remain metabolically active and release factors known as the senescence-associated secretory phenotype (SASP) [253–255]. SASP can have both pro- and anti-tumorigenic effects on the tissue landscape [254, 256–260]. Anti-tumorigenic SASP factors can induce senescence in neighboring cells to safeguard against tumor formation, or recruit immune cells for clearance of tumor cells and senescent cells [254, 256, 257, 260, 261]. Pro-tumorigenic SASP factors can facilitate malignant transformation, promote proliferation, and disrupt tissue structure, tissue function, and/or immune activity to create a tumor promoting environment [255, 259]. Adding to this complexity, senescent cells express distinct SASP profiles that depend on multiple variables, including cell-of-origin, tissue-of-origin, and cause of senescence [253, 255]. For example, Ras-induced senescent human fibroblasts secrete greater levels of factors that promote transformation, compared to senescent fibroblasts induced by replicative exhaustion or irradiation [262]. In mice, Ras-induced senescent hepatic cells produce SASP that suppresses hepatocellular tumorigenesis, by increasing cell clearance [263]. In contrast, *Pten*-loss-induced senescent prostate cells release cytokines that generate a pro-tumorigenic environment, characterized by increased immunosuppressive myeloid-derived suppressor cells (MDSC) and decreased lymphocyte activity [264]. In these ways, senescent cells and SASP play a dynamic and complex role in cancer development, progression, and response to treatment. Here, we will review the current evidence on sex differences in senescence, and discuss how these differences might contribute to sex disparities in cancer.

### Sex differences in senescence

Worldwide, women live longer than men [265, 266], and numerous age-associated diseases—including cardiovascular diseases, neurodegenerative disorders, and cancer—exhibit sex differences in presentation, response to treatment, and mortality [267]. Senescent cells and SASP are important mediators of normal and pathological aging phenotypes. Baker et al. presented that the elimination of senescent cells in mouse models attenuates age-associated changes in multiple tissues. Briefly, senescent cell clearance was associated with greater muscle fiber diameter, preserved muscle function, greater fat deposits, and delayed onset of lordokyphosis and cataracts [268]. In a subsequent study, the group further showed that the clearance of senescent cells led to improved structure and function of the kidney and heart, as well as an increased general healthspan, increased lifespan, and increased tumor latency [269]. Recently, Ruhland et al. showed that senescent cells increase local inflammation in both mouse models and human skin, and that SASP-derived cytokines can promote MDSC infiltration to generate a tumor-permissive environment [259]. These studies support that the accumulation of senescent cells and the release of SASP can have significant impacts on the tissue landscape and can result in tissue dysfunction, inflammation, and tumor formation. Accordingly, numerous studies have demonstrated that senescent cells and SASP are major contributors to multiple age-associated pathologies, such as neurofibrillary tangles, atherosclerosis, osteoarthritis, and cancer [254–257, 259–261, 268–274], which also exhibit significant sex differences in incidence and severity. Given the substantial sex differences in aging and age-associated pathologies, and the evidence that senescence-associated changes in tissue homeostasis mediate many of these pathologies, it seems likely that sex differences in senescence could be a contributing factor. However, this has yet to be investigated directly.

**Fig. 3:**
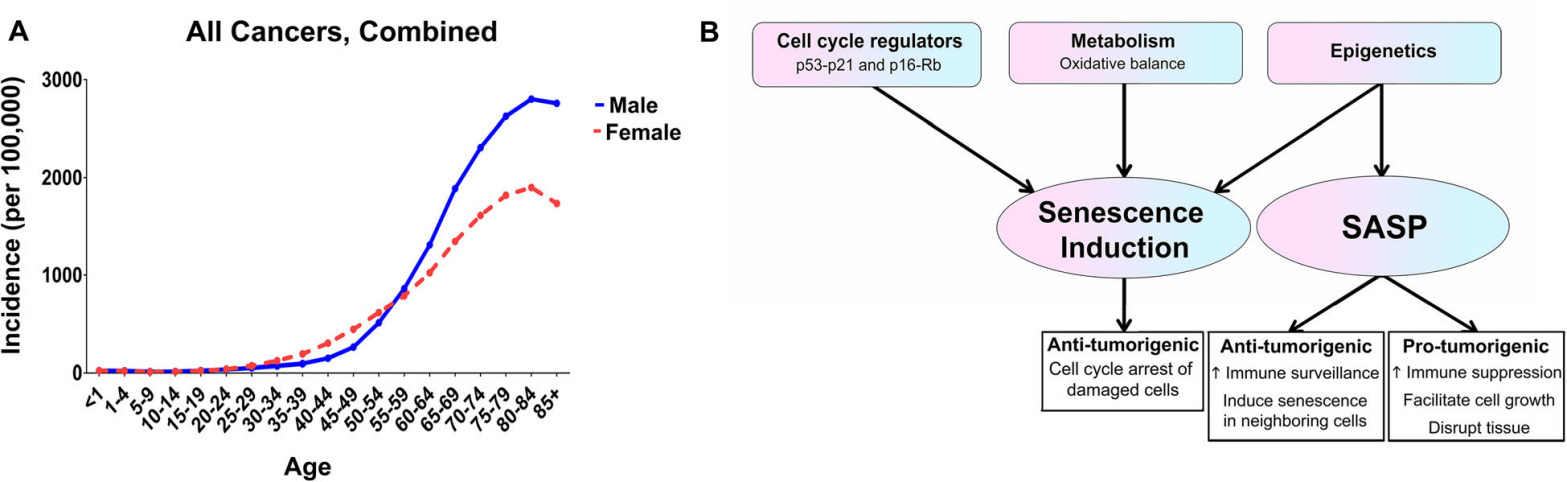
Sex differences in senescence and SASP may contribute to the increasing sex disparity in cancer incidence with age. **a** Cancer incidence increases more steeply in males than in females with age, according to SEER explorer data from 2012–2016^266^. **b** Sex differences in cell cycle regulators, metabolism, and epigenetics can all result in sex differences in senescence activation and SASP. Senescence induction can decrease the risk of transformation in damaged or aged cells, whereas SASP can have both anti-tumorigenic and pro-tumorigenic consequences on the aging tissue. In this way, sex differences in senescence and SASP may contribute to the observed age and sex effects on cancer

Cancer incidence and prevalence rates rise more steeply in males than females with increasing age (Fig. 3) [275]. Whether this is a consequence of sex differences in cellular senescence is unknown. Cells activate senescence as a protective response to DNA-damaging stressors, including telomere attrition, oncogene activation, oxidative stress, and drug/toxin exposure [276–279]. The induction of senescence depends on: (1) the magnitude of genotoxic stress and (2) cellular thresholds for senescence activation; sex may affect both factors. On average, males have shorter telomere lengths than age-matched females, and male cells exhibit faster rates of telomere attrition than female cells [280]. Consequently, male cells may encounter telomere dysfunction sooner than female cells. Furthermore, male cells are more prone to oxidative damage (see “Metabolism” section) and accumulate more somatic mutations than female cells [280]. Together, these studies suggest that males may have a greater risk of exposure to DNA-destabilizing events than females. Finally, there may be sexual dimorphism in toxin metabolism, which can result in sex differences in vulnerability to drug-induced damage. Males and females express distinct activity levels of cytochrome-P450 (CYP450) and UDP-glucuronosyltransferase (UGT) isoforms—two essential enzymatic families involved in drug metabolism [281, 282]. However, the significance of these results to understanding sex differences in drug metabolism and drug-induced cellular damage remains to be elucidated.

While males may exhibit greater DNA damage than females, they may not necessarily activate senescence more frequently. DNA damage activates DNA-damage response (DDR) kinases, such as ATM or ATR [253, 283]. When the damage is determined as irreparable, DDR kinases upregulate the p53-p21 and p16^INK4a^-Rb pathways, resulting in either senescence or apoptosis [253, 283]. Interestingly, under some circumstances, damaged cells remain proliferative despite these protective mechanisms. There may be sex differences in the degree of activation and consequences of these pathways. Malorni et al. found that male and female vascular smooth muscle cells (VSMC) exhibit distinct responses to DNA-damaging stress. After irradiation, male VSMCs more readily underwent apoptosis, whereas female VSMCs expressed senescence characteristics [284]. While sex hormones partially contribute to this sex difference in cell fate [285], cell-intrinsic sex differences are likely involved as well. As described above, there is evidence for sexual dimorphism in cell cycle regulatory pathways (see “p53” section). Notably, after loss of NF1 and p53 function, female astrocytes were more likely to upregulate Rb, p16, and p21—cell cycle regulators involved in senescence—and were less likely to undergo malignant transformation compared to male astrocytes [21, 35]. DNA damage repair, apoptosis, and senescence, each serve as barriers to transformation. However, these pathways are interconnected, and dysfunction in key regulators can affect more than one safeguard. For example, loss of p53 function can compromise both DNA damage repair and pro-apoptotic pathways [286], and activation of compensatory mechanisms such as senescence becomes necessary to avoid transformation. Thus, a male resistance to activating regulators of senescence likely increases their risk for transformation and may contribute to the observed male risk for cancer (Fig. 3).

After senescence induction, the subsequent clearance of senescent cells is important to minimize the accumulation of senescent cells in tissue. As detailed above, the accumulation of senescent cells is associated with multiple age-related diseases, and the elimination of senescent cells leads to increased healthspan, increased lifespan, and the mitigation of age-associated phenotypes and pathologies [254–257, 259–261, 268–274]. A recent study revealed that in both aged wild type mice (30 months) and progeroid mice, males express significantly greater levels of *p16* and *p21* mRNA in the liver, kidney, and spleen than age-matched females. This sex disparity was decreased in wild-type mice at the extremes of old age (35 months): *p16* mRNA levels remained significantly lower in the liver and spleen of females compared to males, while *p21* mRNA levels in the spleen were significantly greater in females than males [287]. These results suggest that males bear a larger load of senescent cells than females throughout aging, which may be influencing the increased male risk for various age-related pathologies, including cancer.

Finally, as mentioned above, senescent cells can also contribute to tumorigenesis through the release of SASP. Given the heterogeneous nature of SASP, sex also may influence the magnitude and/or types of factors released. Yet no studies have investigated sex effects on SASP or how sex differences in SASP affect tumor development or growth.

### Implications for targeting senescence

With increasing age, tissue structure and function are increasingly influenced by the accumulation of senescent cells and the effects of their SASP. This has a significant effect on disease, including cancer. Furthermore, irradiation and chemotherapy, which are mainstays of anti-cancer therapy, can activate therapy-induced senescence (TIS) in cancer cells and neighboring cells. This has been associated with increased therapy-associated toxicity and increased risk for cancer recurrence [288]. For these reasons, there has been growing interest in adjuvant therapies that target senescence to improve treatment response and reduce risk of relapse. These include senescence-inducing drugs, SASP-inhibiting agents, senolytics, senostatics, and synergistic therapies [289, 290]. The development of senescence-targeting drugs is still relatively young, and most agents are undergoing preclinical testing on animal and xenograft models with mixed results. However, there have been recent advances in the field. Particularly, the FDA recently approved palbociclib, a CDK4/6 inhibitor and senescence-inducing agent, for use in ER+/HER2-advanced breast cancer patients [291]. There are also multiple clinical trials studying the efficacy of the combination therapy of navitoclax, a senolytic agent, and chemotherapy against various cancer types. Unfortunately, completed studies show no change in the objective response rate [292]. A common obstacle in the development of senescence-targeted treatments is the lack of a clear understanding of the senescence response and SASP. Further investigation into the mechanisms involved will be integral to formulating more effective treatment strategies, and it is likely that sex-adapted approaches to targeting senescence will be required for the greatest success.

## Immunity

### Immune system in cancer

The immune system plays a significant role in cancer development and progression [293]. This has led to the inclusion of “evading immune destruction” as a hallmark of cancer [294]. There are numerous mechanisms by which tumors avoid immune responses, such as suppression of regulatory T cells [295], down-modulation of antigen processing [296], induction of immune suppressive mediators [297], and promotion of tolerance and immune deviation [298, 299]. As adult females generally mount stronger innate and adaptive immune responses than males [47, 300], sex differences in the immune system likely contribute to the sexual disparity in incidence and mortality associated with certain cancers. Here, we will discuss sex differences in the immune system, and how these differences can lead to sex differences in cancer incidence, mortality, and treatment efficacy.

### Sex differences in the immune system

In the innate immune system, females have antigen-presenting cells (APCs) that are more efficient at presenting peptides than males [301]. As cancer cells modulate antigen-presentation to evade immune destruction, sex differences in APCs and their downstream effector cells can have significant impact on anti-tumor immunity and immunotherapy responses. For example, B7-homolog 1 (B7-H1), a co-signaling molecule expressed abundantly on APCs, which contributes to tumor immune evasion and induces T regulatory cell (Treg) function [302], has been found to be modulated in an estrogen-dependent manner. As a result of reduced Treg function allowing for antitumor immunity, female B7-H1 knock-out mice are more resistant to syngeneic B16 melanoma tumor formation than males [303]. Females also have been shown to have higher numbers and greater phagocytic activity of macrophages and neutrophils than males [304, 305]. Hepatocellular carcinoma (HCC), the most common liver cancer, is 3 to 5 times more likely to develop in males than females [306]. In a mouse model of HCC using a chemical carcinogen, diethylnitrosamine (DEN), it was found that IL-6 production by Kupffer cells (KC), resident liver macrophages, was higher in males than females. IL-6 has been found to be in large concentrations in the tumor microenvironment and is deregulated in cancers [307]. Estrogen reduced circulating IL-6 levels in DEN-treated mice, providing a potential explanation for the reduced liver cancer risk in females [27]. Additionally, there exists a large sex disparity in lung cancer oncogenesis following epithelial *Stat3* deletion in mice with induced mutant *K-ras.* In males, the absence of epithelial STAT3 promotes lung tumorigenesis via enhanced IL-6 [308] signaling and neutrophilic inflammation, which is inhibited in females by estrogen signaling. These studies suggest that estrogen may inhibit inflammatory cytokine secretion by macrophages and neutrophils, reducing cancer risk in females. Conversely, women have a higher incidence of non-small cell lung cancer (NSCLC) [309], but have been shown to have better prognoses than males [29]. It has been suggested that sex differences in NSCLC are due to immune differences, as female NSCLC patients exhibit significantly different immune gene set enrichment compared to males [310].

Within the adaptive immune system several key immune-related genes, such as FOXP3 and CD40L, are located on the X-chromosome. Also, numerous genes expressed in T cells carry the estrogen response element in their promoters, leading to stronger inflammatory and cytotoxic T cell responses in females. These include IFN-γ, IFI6, CX3CL1, CX3CL2, IL-1, IL-5, and IL-16 [311]. The two estrogen receptor subtypes (ERα and ERβ) are expressed on T cells and B cells, suggesting a direct regulatory role of estrogens on these cell types [312]. In humans, CD4+ T cells from females produce higher levels of IFN-γ and proliferate more than male CD4+ T cells. However, male CD4+ T cells have increased IL-17A production compared to females [313]. IL-17A has been shown to have the ability to both increase tumor progression by activating angiogenesis and immunosuppressive activities, and inhibit tumor progression, through recruitment of immune cells into tumors and stimulation of effector CD8+ T cells [314]. Sex differences in T cells of animal models are more pronounced, with CD4+ T cells from females being associated with increased production of IFN-γ and increased responsiveness to IL-12 through STAT-4 activation [312, 313]. These data suggest that the more robust T cell response in females may be beneficial for antitumor immunity. Thus, immunological sex differences in the innate and adaptive immune system, due to both sex hormones and X-linked genes, could contribute to the etiology of sex-related cancer disparities.

### Implications for immunotherapy

**Fig. 4:**
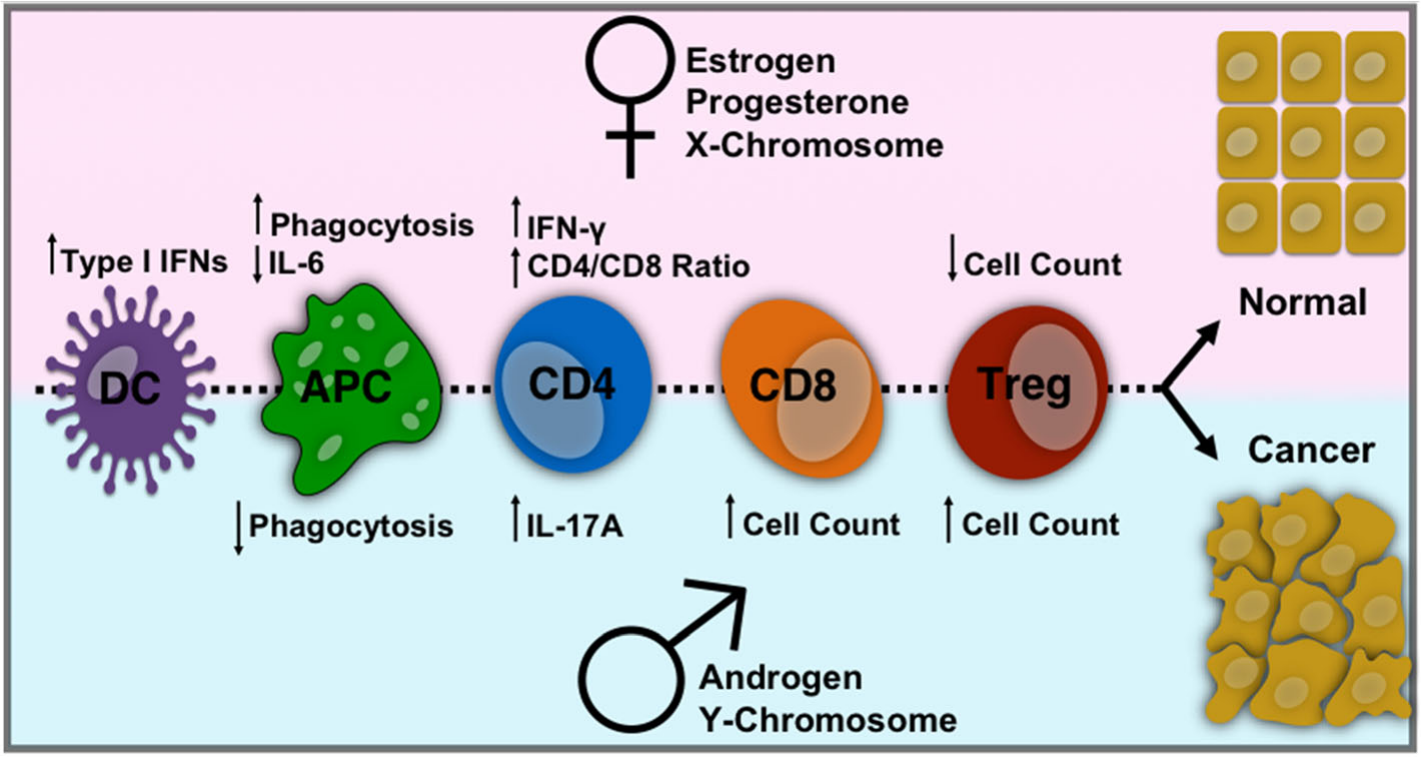
Sex differences in immune cells affecting cancer development. Effects of gonadal hormones and sex chromosomes on cells of the innate and adaptive immune system affecting cancer development. Pink shaded region refers to female differences, and blue shaded region refers to male differences. See text for details

Despite known sex differences in immune responses and functions (Fig. 4), the effect of sex on cancer immunotherapy was not investigated until recently. Successful immunotherapy could enhance the ability of the immune system to mount an effective neo-antigen-specific antitumor response, or stimulate the immune system more generally to mount a vigorous immune response [315]. At least three sites of immune action can be targeted for therapeutic intervention: promoting antigen presentation by dendritic cells (DCs), promoting the production of protective T cell responses, and overcoming immunosuppression in the tumor microenvironment. Estrogens are important regulators of the development and function of DC precursors and DC cell subsets, including plasmacytoid DCs (PDCs), which are a high priority immunotherapy target [316, 317]. Furthermore, females exhibit higher CD4+ T cell counts and higher CD4/CD8 ratios, whereas males have higher numbers of CD8+ and Tregs [47, 318]. Immunotherapies rely on effective antitumor immunity in the tumor microenvironment, which can be achieved by recruiting tumor-infiltrating leukocytes to the site [315]. As leukocyte populations are dramatically affected by sex, antitumor immune responses may display sexual dimorphisms that impact on the efficacy of cancer immunotherapies [319].

Anti-tumor immunotherapy treatments can be antigen-specific, as in monoclonal antibodies, vaccines, and CAR-T cells. There are also antigen-agnostic therapies, such as oncolytic virus therapy and immune checkpoint inhibitors (ICIs), which act as inhibitors of programmed cell death receptor 1 (PD-1) and cytotoxic T-lymphocyte-associated protein 4 (CTLA-4). ICIs have been shown to significantly prolong the overall survival of patients with advanced tumors, by restoring efficacious antitumor immunity [320]. However, these therapies have disproportionately increased efficacy in male compared to female patients [3, 321]. It has also been shown that tumor mutational burden, rather than PD-L1 expression, has a much better predictive power for female response to ICI compared to male response, in lung cancer patients [322]. Furthermore, women experience more immune-related adverse effects compared with men during treatment with anti-PD-1 drugs [323].

The molecular basis for sex differences in response to ICI treatments remains to be fully explored. To date, preclinical studies have demonstrated that the PD-1 ligand (PD-L1)/PD-1 pathway can be regulated by sex steroids [316, 324]. Furthermore, PD-L1 expression has been shown to be modulated by several X-linked micro-RNAs (miRNAs), such as miR-221, miR-222, miR106b, miR-20b, and miR-513 [325]. The X-linked miR424 targets both PD-L1 and CD80, resulting in regulatory control of both the PD-L1/PD-1 and CD80/CTLA-4 pathways [326]. Since mechanisms affecting X-linked genes, such as silencing escape and X-inactivation skewing, could also influence X-linked miRNAs, it is possible that these miRNAs contribute to sex differences in the immune system [327].

As the PD-L1/PD-1 and CD80/CTLA-4 pathways are important targets for ICIs, it is crucial to consider their sexual dimorphism during immunotherapy. For example, as estrogen increases intracellular PD-1 expression [324], female patients may need a higher dose of an ICI to achieve equal immunotherapy efficacy as males. Likewise, as males and females have different numbers and phenotypes of T cell subsets, it may be beneficial to target a specific cell population in each sex to mount effective antitumor immunity. Accordingly, it is critical to consider sex differences in immune functioning and responses when designing cancer preclinical and clinical studies.

## Angiogenesis

### Angiogenesis and cancer

Angiogenesis, the formation of new blood vessels from pre-existing ones, is a hallmark of cancer [294, 328]. Blood vessels are primarily composed of endothelial cells (ECs), which interconnect through tight junctions to form the endothelial lining. Notably, in healthy adults, the vasculature remains quiescent under normal conditions, except for ECs in the female reproductive tract during menstrual cycles and pregnancy [329]. Within the hypoxic tumor microenvironment, abundant pro-angiogenic growth factors are released, including vascular endothelial growth factors (VEGFs), fibroblast growth factors (FGFs), and platelet-derived growth factors (PDGFs) [330–332]. These pro-angiogenic factors bind to pro-angiogenic receptors on ECs, such as VEGF receptor-2 (VEGFR2) [333–336] and integrins [337, 338], and activate downstream signaling pathways like PI3K/Akt [339], leading to endothelial nitric oxide synthase (eNOS) activation [340]. Activated ECs secrete proteases such as matrix metalloproteases (MMPs) to dissolve local basement membrane and extracellular matrix (ECM), allowing ECs to migrate towards the angiogenic stimuli [341]. The signaling events further increase EC proliferation, survival, and differentiation, allowing new capillaries to form and elongate [342, 343]. During angiogenesis, bone marrow-derived endothelial progenitor cells (EPCs) are recruited to the endothelial lining of new blood vessels through vasculogenesis [344]. Other key cell types involved in this angiogenic process include macrophages, pericytes, and fibroblasts [345–348]. In healthy tissue, the process of angiogenesis is tightly regulated by the balance of pro-angiogenic and anti-angiogenic factors, whereas in cancer, this balance is altered to sustain tumor growth, development, and metastasis [342, 349]. Here, we will review current evidence on sex differences in angiogenesis, and discuss how these differences may contribute to sex differences in cancer.

### Sex differences in angiogenesis

**Fig. 5:**
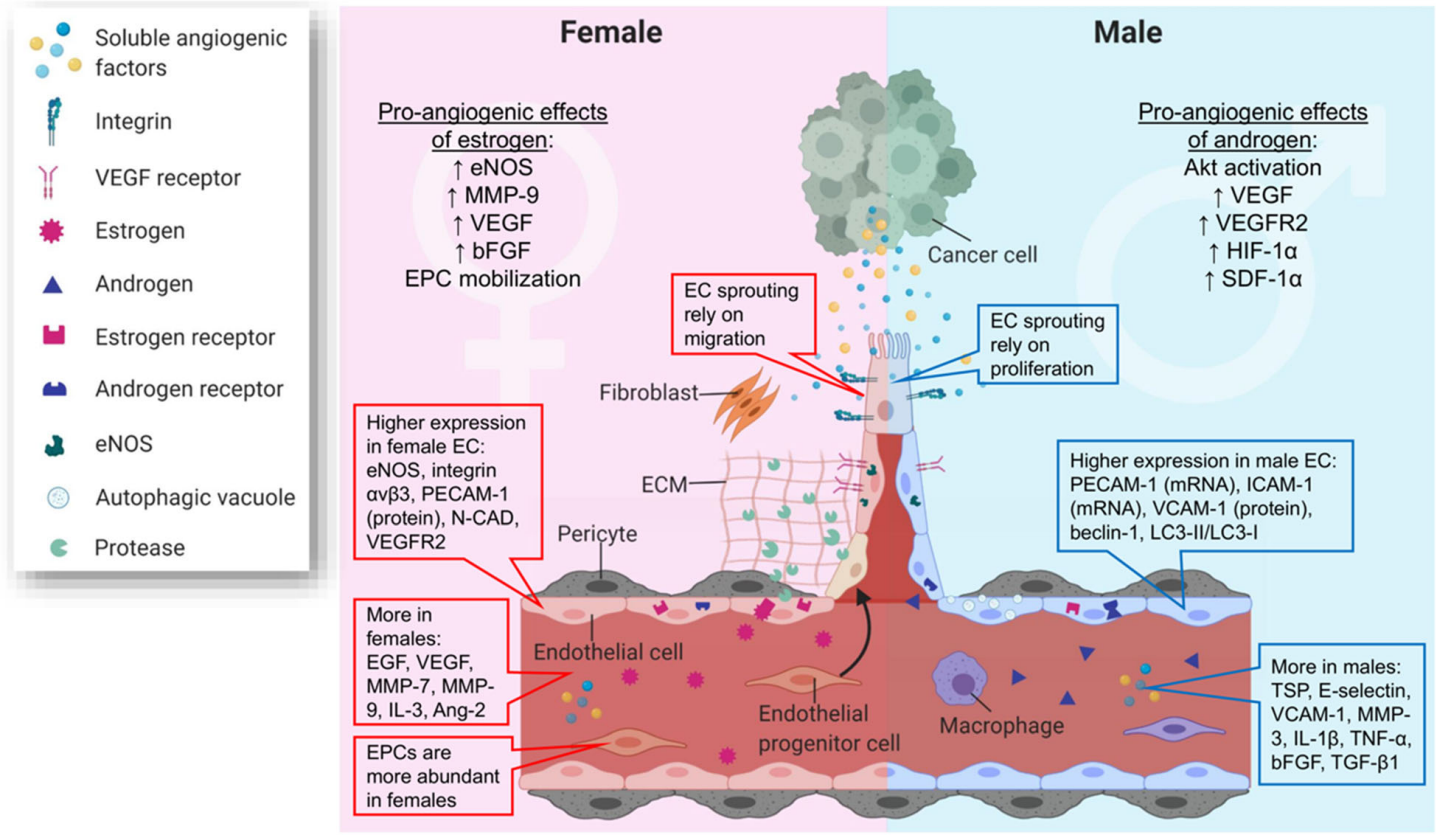
Sex differences in endothelial cells (ECs), endothelial progenitor cells (EPCs), circulating angiogenic factors, and sex hormones contribute to sex differences in tumor angiogenesis. Female and male ECs express different levels of growth factor receptors, integrins, and cell adhesion molecules, which lead to different abilities to migrate and proliferate, and sex-dependent angiogenic mechanisms. Bone marrow-derived EPCs are more abundant in females, which could be a result of sex hormone regulation. Further, estrogen and androgen facilitate angiogenesis through different mechanisms. Finally, females and males have different amounts of circulating pro-angiogenic and anti-angiogenic factors, which may further contribute to the sex differences in tumor angiogenesis

Evidence for sex differences in tumor angiogenesis is limited, with one recent study showing more lymphangiogensis and angiogenesis in lung adenocarcinoma from young female patients than from men [350]. However, several sex differences in EC phenotypes and genotypes, EPC mobilization, circulating angiogenic factors, perivascular tissues, and effects of sex hormones on angiogenesis have been described, thus providing a template for studying sex differences in tumor angiogenesis (Fig. 5).

Female and male ECs present intrinsically different angiogenic phenotypes, and increasing evidence indicates that angiogenesis in males and females may be dependent on different mechanisms. Human umbilical endothelial cells (HUVECs), a macrovascular cell model commonly used for endothelial studies in vitro, when isolated from female newborns, show higher migration ability compared to cells isolated from males [351]. This finding aligns with an ex vivo study that shows faster migration in female macrovascular ECs from rat skeletal muscle compared to male samples [352]. The sexual dimorphism in EC migration ability may be a consequence of female ECs expressing more cell adhesion molecules, including Integrin αvβ3 [352], which promote EC migration through mediating cell-matrix and cell-cell association [337]. Female ECs express higher eNOS and VEGFR2, which may further modulate EC proliferation and migration [351, 353, 354]. Conversely, HUVECs from male newborns are more autophagic than female cells [351]. As EC autophagy has emerged as a critical mechanism facilitating tumor angiogenesis [355], this sex difference in autophagy may contribute to sex differences in tumor angiogenesis, tumor growth, and response to anticancer therapy. Moreover, one study found that female EC sprouting relies on eNOS-mediated migration, while male capillary outgrowth is independent of eNOS but requires cell proliferation [354], suggesting that mechanisms in angiogenesis may be different for male and female ECs. Together, these findings suggest female ECs facilitate angiogenesis mainly through integrin, VEGFR, and eNOS-mediated migration, while male ECs may be less migratory but can still promote angiogenesis through proliferation and other underexplored mechanisms such as autophagy.

In addition to the intrinsic sex differences in ECs, effects of sex hormones, especially estrogen, on angiogenesis have been extensively examined [352, 356, 357]. Studies have identified the expression of ER, progesterone receptor (PR), and androgen receptor (AR) on both ECs and EPCs [351, 358–361]. Androgen was reported to promote proliferation, migration, and angiogenesis in male ECs but not female ECs, both in vitro and in vivo, by the regulation of key angiogenesis-related genes, including HIF-1α and SDF-1α [362]. Estrogen, specifically estradiol (E2), causes rapid eNOS production and increases EC proliferation and migration via ERα and ERβ; although, multiple studies reported that ERα may play a more significant role [357, 359, 361]. E2 can induce bone marrow-derived EPC mobilization via ER binding, followed by PI3K pathway activation, eNOS induction, and FGF-2 production [357]. This E2-mediated EPC mobilization may explain the higher number of EPC (CD34+ VEGFR2+) observed in pre-menopausal women, whereas no significant difference was found between post-menopausal women and age-matched men [363]. E2 has also been found to modestly correlate with hematopoietic progenitor cell counts in women [364]. Interestingly, similar E2-mediated EPC mobilization was also observed in male mice with hindlimb ischemia [356, 365]; additionally, a higher number of EPCs were observed in men with cardiovascular diseases than age-matched women [357], suggesting hormonal regulated angiogenesis may be confounded by cardiovascular diseases. In addition to ECs and EPCs, emerging evidences show that estrogen has an effect on a wide variety of cells in the perivascular environment, including tumor cells, smooth muscle cells, fibroblasts, pericytes, macrophages, and adipose tissue, which can indirectly upregulate angiogenesis by induction of pro-angiogenic factors such as VEGF [366–369]. Together, these estrogen-mediated pro-angiogenic effects align with preclinical and clinical evidence, where a positive correlation between ER expression, angiogenic activity, tumor size, and invasiveness was observed for several cancer types, including breast cancer and lung cancer [350, 370–372].

Sex differences are also evident in the abundance of circulating angiogenic factors. One study of platelet-rich plasma found that pro-angiogenic factors, including epidermal growth factor (EGF), insulin growth factor-1 (IGF-1), PDGF-BB, and VEGF, were significantly higher in women [373]. Conversely, another study of platelet-rich plasma found that PDGF-BB was higher in men and found no sex differences in IGF-1 levels [374]. The same study also found that interleukin-1 beta (IL-1β) and several angiogenic growth factors, including tumor necrosis factor-alpha (TNF-α), basic fibroblast growth factor (bFGF), and transforming growth factor-beta 1(TGF-β1) were higher in males [374]. Further studies do not show a strict pro-angiogenic correlation in females versus males in serum; anti-angiogenic factor angiopoietin-2 and pro-angiogenic factor IL-3 were higher in females, while anti-angiogenic thrombospondin (TSP) and several pro-angiogenic endothelial adhesion molecules (E-selectin, VCAM-1) were higher in males [375]. The same study also showed that MMP-7 and MMP-9 were more abundant in female serum, whereas MMP-3 was higher in male serum [375]. These results indicate a need for meta-analysis to determine if correlations exist between pro- vs. anti-angiogenic factors and sex. Further, these findings suggest a need to uncover why certain growth factors, cytokines, and proteases have sexually dimorphic expression, and whether these sexual dimorphisms translate to differential angiogenic signaling and function.

### Implications for targeting tumor angiogenesis

Since 2004, 14 anti-angiogenic drugs have been approved by the Food and Drug Administration [376]. Primary methods for targeting tumor angiogenesis have relied on antibody inhibitors that block angiogenic growth factors such as VEGF, PDGF, and their signaling pathways [377, 378]. There is already evidence showing sex-dependent response to bevacizumab, a monoclonal antibody that blocks VEGF [379–381]. As increasing evidence demonstrates sex differences in survival outcomes of cancer and responses to therapies [59, 382], preclinical and clinical studies focused on inhibiting angiogenesis should be powered to identify sex differences in treatment response. Furthermore, systematic analysis is necessary in discovering sex-specific molecular targets for anti-angiogenic therapies.

Systems biology offers a promising approach to discover sex-specific molecular targets, and thereby improve anti-angiogenic therapy. Previously, angiogenic receptors on ECs and other perivascular cells have been extensively characterized [336, 353, 383–386] and computationally modeled [387–390]. Computational models, based on mass-action kinetics of the signaling axis, have characterized VEGF–VEGFR binding in both healthy and diseased tissue [391, 392], VEGF spatial distribution in skeletal muscle [393–395], angiogenic sprouting in skeletal muscle [396, 397], and VEGF gradients in peripheral artery disease (PAD) [398]. A recent computational model, which incorporated ex vivo VEGFR concentrations from breast cancer xenografts, predicted that tumors having “high” concentrations of plasma membrane VEGFR1 could be resistant to anti-VEGF drugs (angiogenesis inhibitors) [384, 399]. This systems biology approach, which combines computational modeling and quantitative profiling of the biological system, can similarly predict how sex differences in angiogenic factors and cell receptors can result in differential anti-angiogenic drug response.

## Statistical considerations

In order to optimally detect sex differences in cancer mechanisms like those described above, investigators in the laboratory and the clinic must move beyond simple comparisons of males versus females using *t* tests to evaluate the significance of their differences. Popular analysis strategies for assessing sex differences in cancer are either incorrect or insufficient. Sex differences cannot be inferred from a two-way ANOVA with sex and treatment only; the analysis must include treatment and sex interaction. It is simple and appealing to conduct simple two sample *t* tests to compare treatment within each sex, but this practice is inefficient and insufficient, since no formal inference can be made on the sex difference. Even if an interaction term is incorporated into an ANOVA model, how to derive the contrast to appropriately estimate sex differences is not trivial for most researchers. Appropriate study design, statistical modeling, and tests are required for detecting that interaction. Here, we will briefly consider the necessary components to perform rigorous evaluations of sex effects in cancer.

While completely randomized treatment assignments among a cohort of mice of both sexes is a valid approach to study an interaction effect, deviation from balanced treatment assignments to the two sexes will critically reduce the efficiency of detecting interactions. More effective detection of interactions between treatment and sex, as well as their main effects, can be achieved with a balanced two by two (2 × 2) factorial design. Proper implementation of the factorial design requires randomization and blinding, fundamentals of clinical trial design that are often missing in animal experiments.

**Fig. 6:**
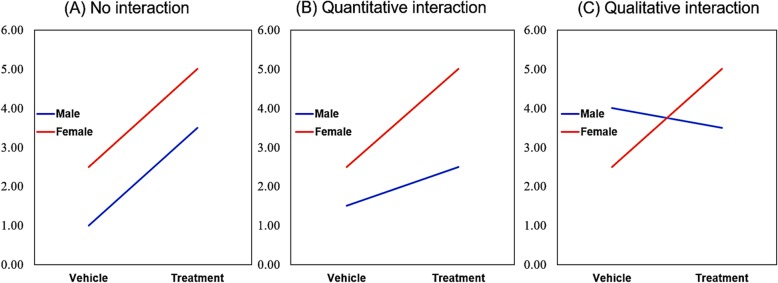
Visualizing statistical interaction. Clinical efficacy outcome of vehicle and treatment is indicated on the *y*-axis. **a** In the presence of the same treatment effect within sex, the sex-specific efficacy lines are parallel, indicating no statistical interaction. In the presence of sex-attributable differences in treatment effect, the two lines will no longer be parallel. In **b**, a quantitative interaction exists between treatment and sex, where treatment improves clinical efficacy in both sexes, but the magnitude of improvement in male is not as great as in female. In **c**, a qualitative interaction exists between treatment and sex, where the treatment benefits female but is detrimental to male, resulting in treatment effects in opposing directions

To illustrate the essential statistical aspects, we evaluate a treatment efficacy outcome, which can be quantitative, binary, or time to event, of two treatments (vehicle vs. treatment) that may differ by sex (female vs. male). Statistical interaction can be visualized by line plots as shown in Fig. 6. Clinical efficacy outcome (assume higher values correspond to better efficacy here) is plotted against treatment, for female and male separately. Two parallel lines indicate the absence of statistical interaction, while two non-parallel lines indicate some interaction exists between treatment and sex. Quantitative interaction (synonymously, non-crossover interaction) exists when treatments benefit both sexes, but with varying magnitude (here females show greater improvement with treatment than male). Qualitative interaction (synonymously cross-over interaction) [400] is present if treatment effects by sex trend in opposing directions (here, treatment is beneficial to female but harmful to male).

In retrospective studies, descriptive statistics can first be generated to summarize an efficacy outcome by combinations of treatment and sex, and sex-attributable effects can be visualized using appropriate plots (Fig. 6). A linear model (or factorial Analysis of Variance (ANOVA) in a balanced design) for a quantitative outcome, a logistic model for a binary outcome, or a Cox proportional hazard model for a survival outcome, can be applied to model the main effects and the interaction effect. The point estimates of treatment efficacy difference between sexes can be estimated, accompanied with 95% confidence interval, though the analysis results are usually regarded as exploratory and are used to generate new hypotheses to prospectively test the existence of a specific type of interaction.

In prospectively designed study on the interaction, we usually conduct two-sided hypothesis testing where an alternative hypothesis (*H*_1_) of unequal treatment efficacy between sexes is tested against a null hypothesis (*H*_0_) of equal treatment effect. In other words, we test on a non-zero difference in treatment efficacy against a zero difference between male and female. Let *δ*_*F*_ and *δ*_*M*_ denote the efficacy difference of treatment versus vehicle in female and male, respectively. The two-sided hypothesis testing can be mathematically written as,

$$ {H}_0:{\delta}_F-{\delta}_M=0\kern0.5em versus\;{H}_1:{\delta}_M-{\delta}_M\ne \kern0.62em 0. $$

Depending on how treatment efficacy is measured, the efficacy difference *δ* can denote the arithmetic difference in the mean efficacy between treatment and vehicle for a quantitatively measured efficacy outcome (Fig. 6), or the odds ratio in logarithm scale for a binary outcome (namely, *δ*_*F*_ and *δ*_*M*_ representing log odds for response in female and male, respectively), or the relative risk (hazard ratio) in logarithm scale for a survival outcome (with *δ*_*F*_ and *δ*_*M*_ representing log hazard for survival in female and male, respectively). For a continuous treatment efficacy outcome *Y*, the sex attributable treatment efficacy difference can be written as,

$$ {\delta}_F-{\delta}_M=\left({\overline{Y}}_{Treatment}^{female}-{\overline{Y}}_{Vehicle}^{female}\right)-\left({\overline{Y}}_{Treatment}^{male}-{\overline{Y}}_{Vehicle}^{male}\right) $$

with $\overline{Y}$denoting the mean treatment efficacy under a treatment for a specific sex, as labeled. The treatment efficacy difference can be estimated and tested against zero by fitting a linear model with the main effects of treatment, sex, and the interaction.

For two-sided hypothesis testing on a qualitative interaction, we specifically test on the presence of opposing directions against the same direction, namely,

$$ {\displaystyle \begin{array}{c}{H}_0:\left({\delta}_F>0\  and\kern0.5em {\delta}_M>0\right)\kern0.5em or\kern0.5em \left({\delta}_F<0\  and\kern0.5em {\delta}_M<0\right)\\ {} versus\kern0.5em {H}_1:\left({\delta}_F>0\kern0.62em and\kern0.5em {\delta}_M<0\right)\kern0.5em or\kern0.5em \left({\delta}_F<0\kern0.62em and\kern0.5em {\delta}_M>0\right)\end{array}} $$

A chi-square heterogeneity test on treatment effect can be used to test quantitative interaction, where the point estimates and standard errors for treatment difference (e.g., hazard ratio or odds ratio) in each sex are estimated and a test statistic is subsequently constructed using Equation (5) and (6) in Gail and Simon [400]. A Gail-Simon likelihood ratio test (GS LR test) can be used to perform specific testing on the presence of a qualitative interaction (using Equation (3a) and (3b) in Gail and Simon [400]) for both a two-sided and a one-sided test. A range test [401] has also been proposed for testing qualitative interaction, which has been reported to have similar power as the GS LR test [400] when only two subgroups (e.g., male and female) are involved.

Sample size needs to be pre-calculated for a prospective study to ensure sufficient power detecting the interaction. For a quantitative outcome, using the sample size equation for normal distribution [402], the total sample size of the study needed to detect the interaction with a type I error rate of α and type II error rate of β (i.e., power = 1-β) can be approximated by,

$$ n=\frac{16^2\left({Z}_{1-\beta }+{Z}_{1-\alpha /2}\right)}{{\left({\delta}_F-{\delta}_M\right)}^2}, $$

with *Z* indicating normal quantiles and *σ*^2^ for the variance of the outcome, and thus *n*/4 for each of the four groups by the combination of treatment and sex. For detecting an interaction between two binary factors in logistic regression for a binary outcome, an online app can be utilized (https://www.dartmouth.edu/~eugened/power-samplesize.php), by specifying the frequencies of outcome, treatment and sex, and the main effect odds ratios of treatment and sex for the binary outcome, as well as the interaction odds ratio [403]. For detecting an interaction between two correlated binary factors in the Cox proportional hazard model for a survival outcome, Equation (6) in Schmoor, et al. [404] can be employed to calculate the sample size; this has been implemented in the R package “powerSurvEpi” (http://CRAN.R-project.org/package=powerSurvEpi), involving the four groups’ prevalence and the hazard ratio of the interaction term. For testing the qualitative interaction using either the GS LR test or the range test, no closed form equation can be used to derive the sample size, and thus simulations have to be used to justify sample size.

Above, we have provided a very brief overview of the statistically relevant issues, focusing on the interaction between two binary factors (treatment and sex). Other methods and test statistics have been proposed in the literature for additional situations, such as treatment interacting with a continuous factor, or more complex, multiple factor interactions.

## Perspectives and significance

Sexual differentiation typically results in two normal and distinct body morphologies and supporting physiologies. This is required for the sex-specific reproductive roles of males and females. The resultant sexual dimorphisms encompass genetic, epigenetic, and metabolic mechanisms at the cellular level, as well as systemic effects on every body system. Consequently, normal growth and aging differ in males and females, as does risk and severity of multiple diseases. Cancer is among those diseases with significant sex differences in risk, treatment response, and outcome. It is imperative that we incorporate the potential for sex differences to significantly impact on the cell and systems biology of cancer. Further, we must accept that these sex differences in cancer biology will affect how males and females respond to therapy, be it standard cytotoxic chemo- and radiation-therapies, immunotherapy, metabolic therapies, or targeted agents. It is essential that our laboratory and clinical research be appropriately powered and analyzed to detect sex effects. Similarly, as the implications of gender identity on health and disease become better defined, these should also be included in paradigms for oncology research and treatment. This is the only way that we can hope to follow through on the promise of precision medicine, to provide the very best care for each and every individual affected by cancer.

## Data Availability

Not applicable

## Abbreviations

GBM: Glioblastoma
BNST/POA: Bed nucleus of the stria terminalis/preoptic area
DNMTs: DNA methyltransferases
eNSCs: Embryonic neural stem cells
IDH: Isocitrate dehydrogenase
iPSCs: Induced pluripotent stem cells
HDACs: Histone deacetylases
lncRNA: Long non-coding RNA
XIC: X inactivation center
XCI: X-chromosome inactivation
LOF: Loss of function
miRNAs: MicroRNAs
α-KG: α-ketoglutarate
2-HG: 2-hydroxyglutarate
ROS: Reactive oxygen species
OGT: O-Linked N-acetylglucosamine transferase
GlcNAc: N-acetylglucosamine
TCA cycle: Tricarboxylic acid cycle
G6PD: Glucose-6-phosphate dehydrogenase
EZH2: Enhancer of zeste-2
DCA: Dichloroacetate
RB: Retinoblastoma protein
EMT: Epithelial to mesenchymal transition
OPC: Oligodendrocyte precursor cells
SVZ: Subventricular zone
LFS: Li-Fraumeni syndrome
NF1: Neurofibromin
CRISPR: Clustered regularly interspaced short palindromic repeats
Cas9: CRISPR-associated protein 9
CNS: Central nervous system
Mdm2: Mouse double minute 2 homolog
SASP: Senescence-associated secretory phenotype
CYP450: Cytochrome-P450
UGT: UDP-glucuronosyltransferase
DDR: DNA-damage response
VSMC: Vascular smooth muscle cells
TIS: Therapy-induced senescence
MDSC: Myeloid-derived suppressor cells
APCs: Antigen-presenting cells
B7-H1: B7-homolog 1
Treg: T regulatory cell
HCC: Hepatocellular carcinoma
DEN: Diethylnitrosamine
KC: Kupffer cells
DCs: Dendritic cells
PDCs: Plasmacytoid DCs
ICIs: Immune checkpoint inhibitors
PD-1: Programmed cell death receptor 1
CTLA-4: Cytotoxic T-lymphocyte-associated protein 4
PD-L1: PD-1 ligand
ANOVA: Analysis of Variance
LR: Likelihood ratio
GS LR test: Gail-Simon likelihood ratio test
WT: Wildtype
SEER: Surveillance, Epidemiology, and End Results
ECs: Endothelial cells
EPCs: Endothelial progenitor cells
VEGFs: Vascular endothelial growth factors
FGFs: Fibroblast growth factors
PDGFs: Platelet-derived growth factors
MMPs: Matrix metalloproteases
VEGFR2: VEGF receptor-2
eNOS: Endothelial nitric oxide synthase
HUVECs: Human umbilical endothelial cells
PECAM1: Platelet-endothelial cell adhesion molecule 1
ER: Estrogen receptor
PR: Progesterone receptor
AR: Androgen receptor
E2: Estradiol
EGF: Epidermal growth factor
IGF-1: Insulin growth factor-1
PDGF: Platelet-derived growth factor
PAD: Peripheral artery disease
BCAAs: Branched-chain amino acids
HK-1: Hexokinase-1
PFK-1: Phosphofructokinase-1
PK-1/2: Pyruvate kinase-1/2
PPP: Pentose phosphate pathway
